# Next-generation sequencing yields the complete organellar genomes of kelp *Lessonia flavicans* (Lessoniaceae, Phaeophyceae) from the Sub-Antarctic ecoregion of Magallanes, Chile

**DOI:** 10.1080/23802359.2019.1688123

**Published:** 2019-11-12

**Authors:** Danilo E. Bustamante, Andres Mansilla, Martha S. Calderon, Sebastian Rosenfeld, Juan P. Rodriguez, Fabio Mendez, Francisco Bahamonde, Karin Gerard, Zambra Lopez, Constanza Ceroni, Veronica Aros, Carolina Perez

**Affiliations:** a Instituto de Investigación para el Desarrollo Sustentable de Ceja de Selva (INDES-CES), Universidad Nacional Toribio Rodríguez de Mendoza, Amazonas, Peru;; b Laboratorio de Ecosistemas Marinos Antárticos y Sub-antárticos (LEMAS), Universidad de Magallanes, Punta Arenas, Chile;; c Instituto de Ecología y Biodiversidad, Punta Arenas, Chile;; d Centro de Investigacion Gaia-Antartica (CIGA), Universidad de Magallanes, Punta Arenas, Chile;; e Centro de Investigación Dinámica de Ecosistemas Marinos de Altas Latitudes (IDEAL), Punta Arenas, Chile;; f Laboratorio Wankara, Universidad de Magallanes, Puerto Williams, Chile

**Keywords:** Chile, kelp, mitogenome, plastid genome, Sub-Antarctic Magellanic ecoregion

## Abstract

The generitype *Lessonia flavicans* Bory is an endemic and important kelp from Sub-Antarctic Magellanic ecoregion that shows affinity to extreme salinity, temperature, and photoperiod conditions. Genomic analysis of *L. flavicans* from Rinconada Bulnes, Punta Arenas, Chile, resulted in the assembly of its organellar genomes. The *L. flavicans* complete mitogenome is 37,226 base pairs (bp) in length and contains 66 genes (GenBank accession number MN561186), the complete plastid genome is 130,085 bp and has 173 genes (MN561187) and the data assembled 8205 bp of the nuclear ribosomal cistron (MN561188). The organellar genomes are similar in structure and content to *L. spicata* (Suhr) Santelices and other Laminariales.

*Lessonia flavicans* is an intertidal to shallow subtidal kelp distributed in the Beagle Channel (Chile), Puerto Deseado (Argentina), and the Falkland Islands (Searles 1978; Mansilla et al. 2014). This species is characterized as having a stipe somewhat treelike cylindrical, branches compressed, blades oval-linear, somewhat denticulated, and yellowish (Searles 1978; Asensi and de Reviers 2009). *Lessonia flavicans* have major ecological roles in the structuring of benthic marine communities and are commercially exploited for the extraction of the phycocolloid alginate (Mansilla et al. 2014). This study characterized the complete organellar genomes of *L. flavicans* from Rinconada Bulnes, Punta Arenas, Chile to determine its genomic structure and genetic relationship as the generitype of the genus *Lessonia*.

DNA was extracted from *L. flavicans* (Specimen Voucher- LEMAS0005) using the NucleoSpin Plant II Kit (Macherey-Nagel, Düren, Germany) following the manufacturer’s instructions. The 150 bp PE Illumina library construction and sequencing were performed by myGenomics, LLC (Alpharetta, GA). The genomes were assembled using default de novo settings in CLC Genomics Workbench version 12.0 (QIAGEN Bioinformatics, Redwood City, CA) and Geneious Prime to close gaps (Biomatters, Ltd, Auckland, New Zealand). The genes were annotated manually using blastx, NCBI ORFfinder, and tRNAscan-SE version 1.21 (Schattner et al. 2005). The *L. flavicans* mitogenome was aligned to other mitogenomes using MAFFT (Katoh and Standley 2013). The phylogenetic analysis was executed with RAxML-NG (Kozlov et al. 2019) using the GTR + gamma model and 1,000 bootstraps. The tree was visualized with TreeDyn version 198.3 at Phylogeny.fr (Dereeper et al. 2008).

The mitogenome of *L. flavicans* is 37,226 bp in length and contains 66 genes. It is A + T rich (66.3%) and includes 25 tRNA (*trnK* and *trnS* occur in duplicate, *trnL* and *trnM* in triplicate), 17 ribosomal proteins, 3 rRNA (*rnl*, *rns*, and *rrn5*), 3 orfs (*orf41*, *orf129*, and orf378), and 18 other genes involved in electron transport and oxidative phosphorylation. The plastid genome of *L. flavicans* is 130,085 bp and contains 173 genes. It is A + T biased (69.2%) and includes 45 ribosomal proteins, 27 tRNA (*trnA*, *trnG*, *trnI*, *trnR*, and *trnS* occur in duplicate, trnM occurs in triplicate), 27 photosystem I and II, 20 ycf, 8 cytochrome b/f complex, 8 ATP synthase, 4 RNA polymerase, 6 rRNA, and 28 other genes. The mitogenome and plastid genome of *L. flavicans* are similar in length, content, and organization to other Laminariales (Chen et al. 2019; Tineo et al. 2019; Zheng et al. 2019).

Phylogenetic analysis of the *L. flavicans* mitogenome resolved it in a fully supported clade with *L. spicata* (Figure 1). The mitogenome of *L. flavicans* differed in pairwise distance from *L. spicata* by 3.8%. The plastid genome of *L. flavicans* differed from *L. spicata* by 1.4%. The evolutionary relationship of the generitype *L. flavicans* confirms that Lessoniaceae is closely allied with Laminariaceae (Tineo et al. 2019).

**Figure 1.**
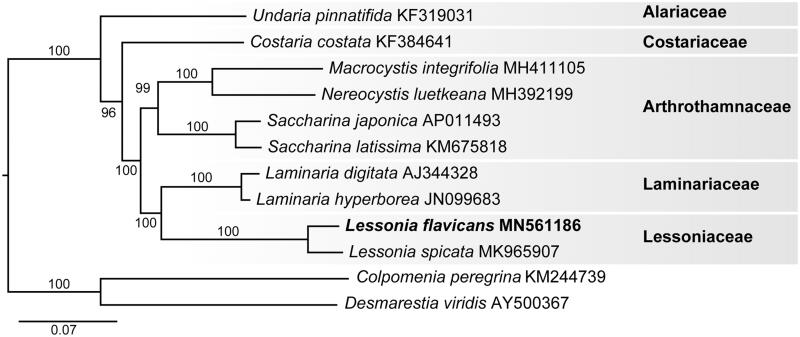
Maximum-likelihood phylogram of *Lessonia flavicans* (MN561186) and related Laminarialean mitogenomes. Numbers along branches are RaxML bootstrap supports based on 1000 replicates. The legend below represents the scale for nucleotide substitutions.
